# Pharmacological treatment after a first myocardial infarction in Sweden—long-term adherence and prognosis

**DOI:** 10.1093/ehjqcco/qcag050

**Published:** 2026-03-26

**Authors:** Maria Sakalaki, Michael Fu, Aldina Pivodic, Annika Rosengren, Lena Björck

**Affiliations:** Department of Molecular and Clinical Medicine, Institute of Medicine, Sahlgrenska Academy, University of Gothenburg, Medicinaregatan 3, våning 5, 405 30 Gothenburg, Sweden; Department of Medicine, Geriatrics and Emergency Medicine, Sahlgrenska University Hospital/Östra, Diagnosvägen 11, 416 85 Gothenburg, Sweden; Department of Molecular and Clinical Medicine, Institute of Medicine, Sahlgrenska Academy, University of Gothenburg, Medicinaregatan 3, våning 5, 405 30 Gothenburg, Sweden; Department of Medicine, Geriatrics and Emergency Medicine, Sahlgrenska University Hospital/Östra, Diagnosvägen 11, 416 85 Gothenburg, Sweden; Department of Clinical Neuroscience, Institute of Neuroscience and Physiology, Sahlgrenska Academy, University of Gothenburg, 405 30 Gothenburg, Sweden; APNC Sweden, GoCo Health Innovation City, Entreprenörsstråket 10, 431 53 Mölndal, Sweden; Department of Molecular and Clinical Medicine, Institute of Medicine, Sahlgrenska Academy, University of Gothenburg, Medicinaregatan 3, våning 5, 405 30 Gothenburg, Sweden; Department of Medicine, Geriatrics and Emergency Medicine, Sahlgrenska University Hospital/Östra, Diagnosvägen 11, 416 85 Gothenburg, Sweden; Department of Molecular and Clinical Medicine, Institute of Medicine, Sahlgrenska Academy, University of Gothenburg, Medicinaregatan 3, våning 5, 405 30 Gothenburg, Sweden; Department of Medicine, Geriatrics and Emergency Medicine, Sahlgrenska University Hospital/Östra, Diagnosvägen 11, 416 85 Gothenburg, Sweden

**Keywords:** Secondary prevention, Myocardial infarction, Pharmacological treatment

## Abstract

**Aims:**

Optimizing pharmacological treatment for secondary prevention after myocardial infarction (MI) is crucial to prevent new events, but few have reported data over a long-term follow-up. The aim was to study long-term follow-up of guideline-directed medical therapy (GDMT) as a part of secondary prevention after MI.

**Methods and results:**

Patients aged 18–84 years, hospitalized with a first acute MI between 2006 and 2022 were identified through the Swedish National Inpatient Register linked to the Prescribed Drug Register. GDMT was defined as antiplatelet/oral anticoagulant therapy (OAC), lipid lowering therapy, beta-blockers and angiotensin converting enzyme inhibitors (ACEi)/angiotensin receptor blockers (ARB). Time-updated survival analyses were used to investigate the relationship between GDMT and mortality and/or recurrent MI. A total of 96 272 individuals with acute MI were included (29.3% women). At baseline 48.4% had hypertension, 18.8% diabetes mellitus and 13.5% heart failure. Use of GDMT decreased markedly during the first year after MI. During median follow-up of 5.6 years 32.9% died. Medication with antiplatelet/OAC, lipid lowering treatment and ACEi/ARB were associated with lower risk of all cause-mortality or recurrent MI hazard ratio (HR) 0.76 [95% confidence interval (CI) 0.74–0.78], HR 0.68 [95% CI 0.67–0.70], HR 0.81 [95% CI 0.79–0.83] respectively (all *P* < 0.0001). Use of beta-blockers was associated with a slightly increased risk, HR 1.04 [95% CI 1.02–1.07], *P* = 0.0005.

**Conclusions:**

The use of all GDMT is associated with lower risk for all-cause mortality and recurrent myocardial infarction. Long-term adherence of GDMT is suboptimal and there is a potential for further improvement.

## Structured graphical abstract

**Structured graphical abstract:**
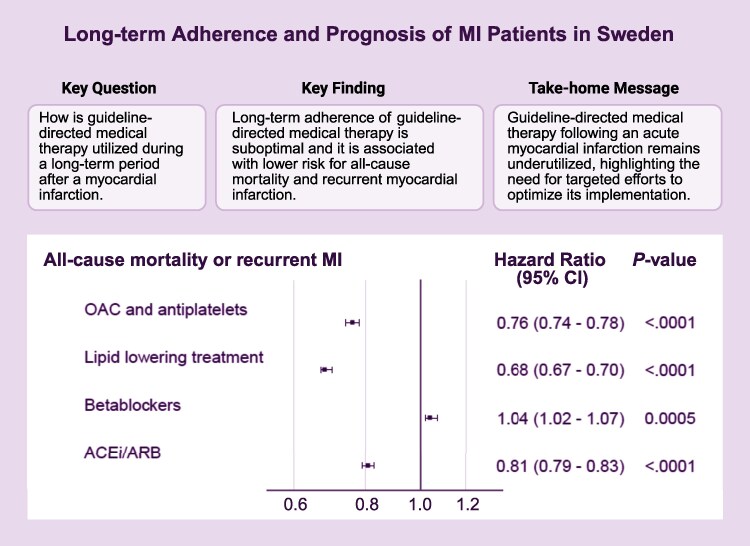

## Background

Secondary prevention including lifestyle changes and guideline-directed medical therapy (GDMT) after an acute myocardial infarction is crucial to prevent recurrent events and prolong survival. European guidelines recommend the use of several drugs to this end.^1^ Antiplatelet, lipid lowering therapy, angiotensin converting enzyme inhibitors (ACEi) or angiotensin II receptor blockers (ARB) and beta-blockers are the four main pharmacological classes that are included in the secondary preventive regime. Oral anticoagulants (OACs) are used in place of, rather than interchangeably with, antiplatelet therapy in clinical situations where OAC treatment is indicated, such as atrial fibrillation. Antiplatelet and lipid lowering treatment have a strong recommendation, and the guidelines advocate prescription to all patients after a myocardial infarction. ACEi or ARB should be given to all patients with either heart failure symptoms, ejection fraction <40%, hypertension, diabetes mellitus and/or chronic kidney disease.^1^ The use of beta-blockers is recommended on the basis of older studies.^2^ However, recent data shows no protective effect of long-term beta-blocker treatment in myocardial infarction patients undergoing early angiography and with a preserved left ventricular ejection fraction.^3^ Available data about the use and effect of pharmacological treatment for secondary prevention after a myocardial infarction are inconsistent. Study follow-up is often shorter than 10 years, mainly showing that continuation to drug treatment decreases with time.^4–8^

Several determinants to discontinuation of drugs after a myocardial infarction have been proposed. Earlier studies have shown less prescribed guidelines recommended medication for secondary prevention in women.^9–11^ Socioeconomic factors, ethnicity, and emotional distress can affect medical adherence negatively after a myocardial infarction,^12–14^ while social support and coherence increase the probability of optimal secondary prevention.^15^

Long-term pharmacological therapy following myocardial infarction is essential and serves as a cornerstone in reducing mortality and limiting the progression of ischaemic heart disease.^16–18^ However, the long-term use and effects of GDMT after a first myocardial infarction remain largely unexplored. We used nationwide register data to examine long-term follow-up of secondary preventive GDMT after a myocardial infarction and to which extent there is an association with outcome.

## Method

### Study design and patients

In this register study the Swedish National Inpatient Register (IPR) was used to identify individuals aged 18–84 years hospitalized with a first myocardial infarction between 1 January 2006 and 31 December 2022. We used the Swedish Prescribed Drug Register (SPDR) for information on dispensed medication after a myocardial infarction. Drugs in the register are categorized according to the Anatomic Therapeutic Chemical (ATC) classification. Four classes of drugs were analyzed in this study: antiplatelet and OAC, lipid lowering therapy, ACEi or ARB and beta-blockers (see Supplementary material online, *Table S1*). Linkage between registers was done through the personal identification number (PIN) issued to all Swedish citizens.

### Definitions

The drugs prescribed for medical treatment after an acute myocardial infarction was time-updated in the analyses starting from the discharge date. Patients who retrieved their medication within 6-month period but did not obtain a refill within the subsequent 6 months were considered to have discontinued treatment. In Sweden, patients typically receive a 3-month supply of medication at each refill. To allow for potential delays—such as hospitalizations, medication adjustments, or temporary lapses due to forgetfulness—a 6-month period was selected to provide a sufficient window for medication refills. Therefore, the exposure assessment interval was updated every third month reflecting use over the prior 6 months. The end of observation was the studied outcome, death, migration date or 31 December 2022.

Marital status was divided into five categories: married/cohabiting, not married, divorced, widowed and unknown. Income was the disposable yearly income of the household adjusted for the consumer price index and was stratified into quintiles, from Q1 (lowest) to Q5 (highest), before applying exclusion criteria. Country of birth was divided into two groups: Nordic and non-Nordic. Educational level had three categories: <10 years (compulsory school attendance), 10 to 12 years (upper secondary school) and >12 years (university/college). Missing data of these variables were considered as an own category in the adjustment analyses.

### Outcome definition

The international classification of disease ICD-9 and ICD-10 were used to define myocardial infarction (410 for ICD-9, and I21 for ICD-10), comorbidities and non-fatal outcomes (see Supplementary material online, *Table S2*). The National Cause of Death Register was used to identify all-cause mortality and cardiovascular mortality outcome. Death due to myocardial infarction was registered as the ICD code for myocardial infarction.

### Registers

Sweden operates a predominantly publicly funded healthcare system, with subsidized costs for hospitalizations, medical procedures, and prescription medications. The IPR contains comprehensive data on all hospital discharges nationwide, including primary and secondary diagnoses. The IPR is part of the Swedish National Patient Register, which was established by the National Board of Health and Welfare and has had full national coverage since 1987.^19^

Data on social variables such as marital status, country of birth, income and education was collected from the Longitudinal Integration Database for Health Insurance and Labour Market (LISA) register. LISA contains data from 1990 onwards and includes individuals aged 15 years and older who are registered in the Swedish population. The database is updated annually.^20^ The National Cause of Death Register contains data of deaths of the population of Sweden since 1961 and was used for information on mortality dates.^21^ The SPDR was established 1 July 2005, and includes the PIN of every individual, allowing linkage between data on dispensed drugs and other registers.^22^ Subjects using an ‘Apodos’ services were excluded to avoid bias that would have overestimated treatment adherence. Apodos is a service provided from Swedish pharmacies to facilitate drug dispensation, by delivering small packages with drugs in correct dosage, date and time for intake.

### Statistical analyses

Continuous variables are presented as means, standard deviation, or median, and interquartile range, and categorical variables by frequency and percentages.

For testing differences between two groups Fisher’s exact test was used for dichotomous variables, Mantel-Haenszel chi-square trend test for ordered categorical variables, chi-square test for non-ordered categorical variables and for continuous variables Mann–Whitney U-test.

Event rates were calculated as number of events divided by the total number of follow-up years, expressed by 100 person years. Lower and upper 95% confidence limits were estimated using exact Poisson limits.

Time to event analyses were performed using time-updated adjusted Cox regression models, incorporating the four classes of drugs as time-updated variables. Medication use was assessed every 3 months, reflecting the usage during the 6 months period, and this process continued until the event occurred or censoring. Hazard ratios (HR) with 95% confidence interval (CI) are presented. Adjustments were made for the following confounders: age, sex, separately for each medication group (unless main effect variable), calendar year, Nordic/non-Nordic country, marital status, education, income, diabetes, hypertension, heart failure, atrial fibrillation, stroke, chronic respiratory disease/asthma, peripheral vascular disease, kidney failure, congenital heart disease, cancer and valvular disease.

A complementary post-hoc analysis using inverse probability treatment weighting (IPTW) of the study’s main results was performed. Propensity scores were calculated based on age, country of birth, sex, marital status, education, income quantile, diabetes, hypertension, heart failure, atrial fibrillation, stroke, chronic respiratory disease, peripheral vascular disease, kidney failure, congenital heart disease, cancer, and valvular disease.

To evaluate the impact of cumulative medication use, analyses were repeated for different durations: <1 year, 1–<5 years, 5–<10 years, and ≥10 years, each compared with no medication use. Time to discontinuation of medications was also analyzed using age- and sex-adjusted Cox regression models to assess the predictive effect of baseline characteristics on the outcome. A hazard ratio ≥1.20 or ≤0.80 was considered clinically relevant in these analyses, to avoid over interpretation of trivially small effects in a very large cohort. All tests were two-tailed. *P*-values <0.05 were considered significant. All analyses were performed by using SAS® Software v9.4 (SAS Institute Inc., Cary, NC, USA).

## Results

### Study population

**Figure 1:**
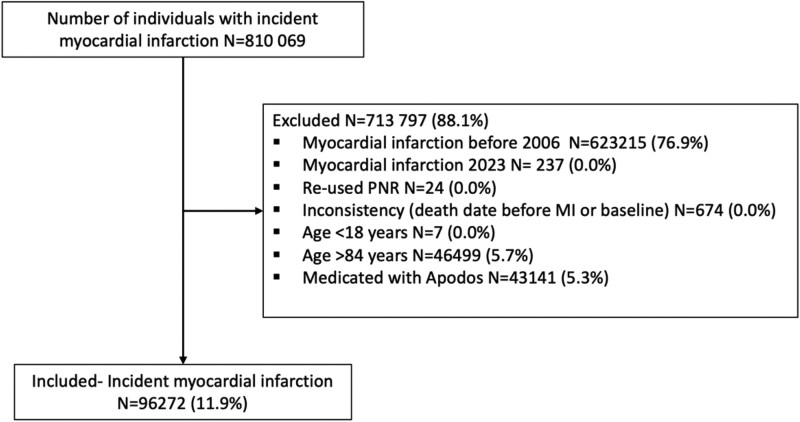
Study flow chart. MI, myocardial infarction; PNR, personal number.

After excluding patients with pre-packaged medications (Apodos) (*n* = 43 141), 96 272 individuals were included in the study (*Figure 1*). The mean age was 67.5 ± 10.6 years and women accounted for 29.3%. At baseline, the most common pre-existing diagnoses were hypertension (48.4%), diabetes mellitus (18.8%), heart failure (13.5%), atrial fibrillation (11.3%) and cancer (16.0%). Most of the patients were born in a Nordic country (87.9%) and were married or cohabiting (57.0%) (*Table 1*). Patient demographics, stratified by first-year medication discontinuation, are shown in Supplementary material online, *Table S3*. The groups demonstrated comparable baseline characteristics.

**Table 1:** Baseline characteristics of patients hospitalized with a first myocardial infarction 2006–2022

Variable	Total *n* = 96 272	Male *n* = 68 036	Female *n* = 28 236	*P*-value
***Patient demographics and*** ***socioeconomic status***				
Age (years)	67.5 ± 10.6 69 (18–84) *n* = 96 272	66.6 ± 10.6 67 (18–84) *n* = 68 036	69.6 ± 10.3 71 (18–84) *n* = 28 236	<0.0001
Age category (years)				<0.0001
<65 years	35 338 (36.7%)	27 317 (40.2%)	8021 (28.4%)	
65–74 years	32 408 (33.7%)	22 870 (33.6%)	9538 (33.8%)	
75–84 years	28 526 (29.6%)	17 849 (26.2%)	10 677 (37.8%)	
Country of birth				<0.0001
Nordic countries	84 633 (87.9%)	59 106 (86.9%)	25 527 (90.4%)	
Non-Nordic countries	11 611 (12.1%)	8907 (13.1%)	2704 (9.6%)	
Missing	28	23	5	
Marital status				<0.0001
Married/Cohabiting	54 831 (57.0%)	41 288 (60.7%)	13 543 (48.0%)	
Not married	14 919 (15.5%)	11 591 (17.0%)	3328 (11.8%)	
Divorced	17 793 (18.5%)	11 878 (17.5%)	5915 (20.9%)	
Widowed	8641 (9.0%)	3220 (4.7%)	5421 (19.2%)	
Unknown	88 (0.1%)	59 (0.1%)	29 (0.1%)	
Education (years)				<0.0001
<10	31 965 (33.7%)	22 138 (33.0%)	9827 (35.4%)	
10–12	41 821 (44.1%)	29 651 (44.2%)	12 170 (43.9%)	
>12	21 088 (22.2%)	15 351 (22.9%)	5737 (20.7%)	
Missing	1398	896	502	
Income				<0.0001
Q1 (lowest)	11 370 (11.8%)	6362 (9.4%)	5008 (17.8%)	
Q2	13 766 (14.3%)	8143 (12.0%)	5623 (19.9%)	
Q3	18 557 (19.3%)	13 127 (19.3%)	5430 (19.3%)	
Q4	23 900 (24.9%)	17 680 (26.0%)	6220 (22.1%)	
Q5 (highest)	28 579 (29.7%)	22 656 (33.3%)	5923 (21.0%)	
Missing	100	68	32	
***Medical history before incident MI diagnosis***				
Diabetes	18 133 (18.8%)	12 699 (18.7%)	5434 (19.2%)	0.037
Hypertension	46 566 (48.4%)	31 304 (46.0%)	15 262 (54.1%)	<0.0001
Heart failure	12 949 (13.5%)	8739 (12.8%)	4210 (14.9%)	<0.0001
Atrial fibrillation	10 880 (11.3%)	7537 (11.1%)	3343 (11.8%)	0.0007
Stroke	6464 (6.7%)	4465 (6.6%)	1999 (7.1%)	0.0037
Chronic respiratory disease/asthma	10 198 (10.6%)	5747 (8.4%)	4451 (15.8%)	<0.0001
Peripheral vascular disease	7297 (7.6%)	4956 (7.3%)	2341 (8.3%)	<0.0001
Kidney failure	5058 (5.3%)	3623 (5.3%)	1435 (5.1%)	0.13
Adult congenital heart disease	297 (0.3%)	192 (0.3%)	105 (0.4%)	0.025
Cancer	15 421 (16.0%)	10 520 (15.5%)	4901 (17.4%)	<0.0001
Valvular disease	4840 (5.0%)	3192 (4.7%)	1648 (5.8%)	<0.0001
***Medications before incident MI diagnosis***				
OAC and antiplatelets	26 300 (27.3%)	18 211 (26.8%)	8089 (28.6%)	<0.0001
Lipid lowering treatment	22 810 (23.7%)	15 842 (23.3%)	6968 (24.7%)	<0.0001
ß-blockers	25 998 (27.0%)	16 702 (24.5%)	9296 (32.9%)	<0.0001
ACEi/ARB	32 578 (33.8%)	22 227 (32.7%)	10 351 (36.7%)	<0.0001

Data are presented as mean ± standard deviation, median (range) and number of observations, or number (%).

ACEi, angiotensin converting enzyme inhibitor; ARB, angiotensin receptor blocker; OAC, oral anticoagulant.

### Pharmacological treatment and discontinuation

**Figure 2:**
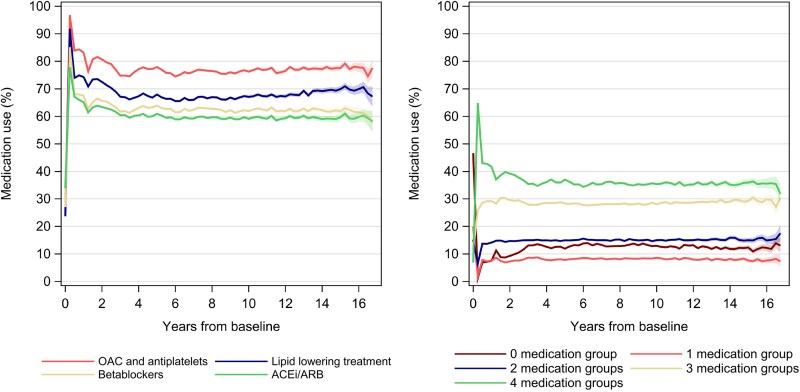
Medication use over time in patients with a first myocardial infarction 2006–2022. ACEi, angiotensin converting enzyme inhibitor; ARB, angiotensin receptor blocker; OAC, oral anticoagulant.

Some patients were already treated with ACEi/ARB (33.8%), beta-blockers (27.0%), antiplatelets/OAC (27.3%) and lipid lowering treatment (23.7%) before the index myocardial infarction, (*Table 1*). After the myocardial infarction, filled prescriptions of ACEi/ARB, beta-blockers, lipid lowering treatment and antiplatelets/OAC increased to 67.1%, 68.0%, 74.0% and 83.9% respectively (*Figure 2*). Following an initial increase, the use of all classes of pharmacological treatments declined after the first 2 years post-myocardial infarction, plateauing thereafter. Many patients retained at least one of the four drug classes consistently throughout the study period; however, approximately one in six remained without any of the drugs.

### Associations with all-cause mortality, recurrent myocardial infarction and cardiovascular mortality

During follow-up 31 652 died [32.9%, event rate 5.10 per 100 person years (95% CI 5.05–5.16)], of which 16 873 [17.5%, event rate 2.72 per 100 person years (95% CI 2.68–2.76)] died due to cardiovascular disease. A total of 17 128 persons [17.8%, event rate 3.33 per 100 person years (95% CI 3.28–3.38)] had a recurrent myocardial infarction. A composite outcome of all-cause mortality or recurrent myocardial infarction occurred in 42 202 [43.8%, event rate 8.21 per 100 person years (95% CI 8.14–8.29)] individuals. Median follow-up time was 5.61 years (interquartile range 1.85–10.6) for all-cause and cardiovascular mortality, and 4.01 years (interquartile range 0.51–9.24) for recurrent myocardial infarction (see Supplementary material online, *Table S4*).

**Figure 3:**
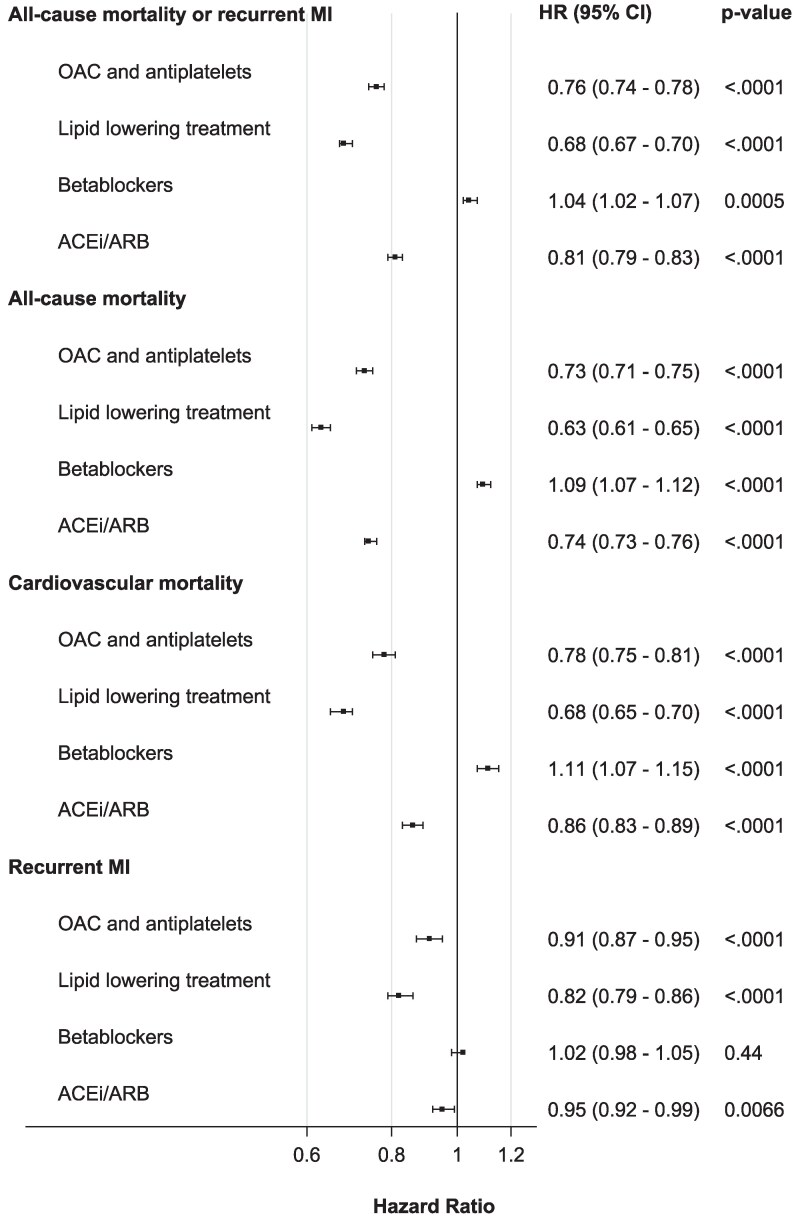
Forest plot of adjusted effects of time-updated medications for all-cause mortality, recurrent myocardial infarction and cardiovascular mortality in individuals with first myocardial infarction 2006–2022. ACEi, angiotensin converting enzyme inhibitor; ARB, angiotensin receptor blocker; MI, myocardial infarction; OAC, oral anticoagulant.

Treatment with lipid lowering treatment, antiplatelets or OAC and ACEi or ARB was associated with a lower risk for the composite outcome of all-cause mortality or recurrent myocardial infarction following a first myocardial infarction event, HR 0.68 (95% CI 0.67–0.70) for lipid lowering treatment, HR 0.76 (95% CI 0.74–0.78) for antiplatelets or OAC and HR 0.81 (95% CI 0.79–0.83) for ACEi/ARB (*Figure 3*). Use of beta-blockers was associated with a slightly higher risk of all-cause mortality or recurrent myocardial infarction by 4%, [HR 1.04 (95% CI 1.02–1.07), *P*-value = 0.0005]. When studying the two outcomes separately, treatment with lipid lowering treatment, antiplatelets/OAC or ACEi/ARB was associated with lower risk of all-cause mortality, while beta-blocker treatment with slightly higher risk, [HR 1.09 (95% CI 1.07–1.12)] for all-cause mortality. Similar associations were seen when studying cardiovascular mortality and recurrent myocardial infarction separately, but less markedly for recurrent myocardial infarction, and with a neutral association for treatment with beta-blockers (*Figure 3*). Results from the complementary analyses using IPTW method were well-corresponding to the main results applying adjustments (see Supplementary material online, *Figure S1*).

A combination of all three drug groups, antiplatelets/OAC, lipid lowering treatment and ACEi/ARB, was associated with additionally lower risk for all four outcomes studied (see Supplementary material online, *Table S5*). Adding a fourth medical group of beta-blockers did not further lower the risk for any outcome.

In addition, the cumulative significance of persistence to medical treatment (<1 year, 1–<5 years, 5–<10 years and ≥10 years) for all-cause mortality, cardiovascular mortality and recurrent myocardial infarction was examined for each drug group. Longer treatment with antiplatelets or OAC was associated with lower risk for all outcomes. ACEi/ARB and lipid lowering treatment showed varying results for longer medication persistence and different outcomes. Continuous use of beta-blockers was associated with higher risk with time for all outcomes, except for recurrent myocardial infarction (see Supplementary material online, *Tables S6.1*  to  *S6.4*).

### Covariates for discontinuation

**Figure 4:**
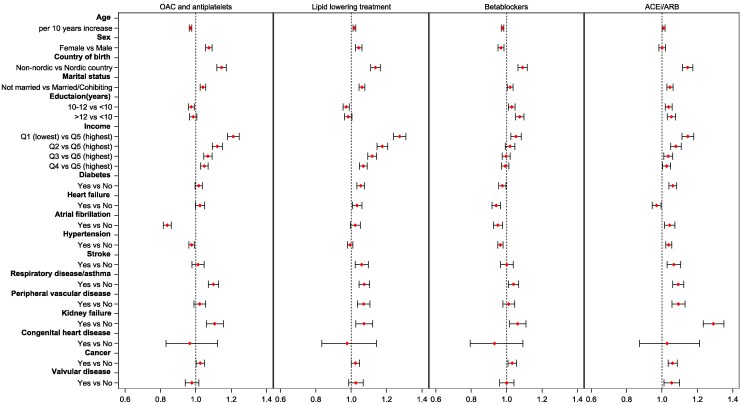
Forest plot of age- and sex-adjusted Cox regression for analyses for time to discontinuation of medications. ACEi, angiotensin converting enzyme inhibitor; ARB, angiotensin receptor blocker; OAC, oral anticoagulant.

The effects on discontinuation by different covariates as sex, socioeconomic variables and comorbidities are shown in *Figure 4* for each drug group. Income was associated with 27% higher risk for discontinuing lipid lowering treatment [HR 1.27 (95% CI 1.24–1.31), *P*-value <0.0001] and 21% higher risk for discontinuing antiplatelets/OAC [HR 1.21 (95% CI 1.18–1.24), *P*-value <0.0001], respectively.

Furthermore, having other comorbidities affected the risk for discontinuation. This was especially noticeable for having kidney failure, which was associated with higher risk for discontinuation for all drug groups, in particular ACEi/ARB, [HR 1.29 (95% CI 1.23–1.35), *P*-value <0.0001].

## Discussion

In this study on the long-term continuation of pharmacological treatment, we found that the use of GDMT was high during the first 1 to 2 years after discharge but then decreased to reach a plateau of underutilization for all drug groups. Simultaneously, results showed that treatment with GDMT was associated with lower mortality risk and less recurrent myocardial infarction.

Our findings are in line with the annual report of 2023 from SWEDEHEART (The National Quality Registry for Enhancement and Development of Evidence-Based Care in Heart Disease) and European survey on cardiovascular risk factor control EUROASPIRE V, showing high use of medical treatment for all four groups during first period after discharge from hospital.^23,24^ Use seems to stabilize after the initial decline, which might reflect that those with successful early adherence and tolerance of the treatment maintain their medication use. WHO addressed the issue of adherence to long-term therapies in a comprehensive report, stating that adherence is affected by patient, medical and condition related factors. Other reasons are socio-economic factors and the health system, with the latter including issues such as insufficient support from health providers and poor communication.^25^ Time to first out-patient visit might also influence the adherence to pharmacological treatment,^8^ where a shorter time from discharge to first follow-up visit can increase medical adherence. Similar findings of suboptimal use of guideline-recommended OAC and antiplatelet therapies were reported in a recent study evaluating pharmacological treatment within 40 days after discharge following hospitalization for acute MI.^26^ The authors emphasized the importance of close collaboration between hospital and primary care providers. In sum, this demonstrates the importance of long-term follow-up and support in secondary prevention.

In this study the follow-up time were more than 10 years, but a median follow-up of 5.61 years and a wide interquartile range across the whole population. Although follow-up time varied substantially, the extended inclusion criteria allowed us to capture long-term trends in medication persistence and outcomes in a real-world setting. While only a subset of patients had more than 10 years of follow-up, the large sample size and time-updated analyses provide robust insights into persistence and discontinuation patterns over time.

Moreover, we observed associations of treatment with antiplatelets/OAC, ACEi/ARB and lipid lowering treatment with lower risk of all-cause mortality, cardiovascular mortality and recurrent myocardial infarction, similar to what has been shown in other studies.^9,27,28^ However, this association of benefit was not evident for treatment with beta-blockers, rather than a small but significant higher risk. Adding use of beta-blockers to the other three medical groups showed that the risk for mortality and recurrent myocardial infarction was not further reduced. Previous studies have shown inconsistent results on the benefit of beta-blockers after an acute myocardial infarction and especially in those with no heart failure.^29–31^ Therefore, the early initiation of oral beta-blockers after an acute myocardial infarction with preserved ejection fraction was recently investigated in a randomized, registry-based and prospective study, Beta-Blockers after Myocardial Infarction and Preserved Ejection Fraction (REDUCE-AMI). This trial demonstrated no difference of the primary composite end-point of all-cause mortality and new myocardial infarction between those with beta-blocker treatment and those without [HR 0.96 (95% CI 0.79–1.16), *P*-value <0.064] and similar incidence of safety endpoint.^3^ Furthermore, the ABYSS trial (the assessment of β-blocker interruption 1 year after an uncomplicated myocardial infarction on Safety and Symptomatic cardiac events requiring hospitalization) showed that patients with a history of myocardial infarction and left ventricular ejection fraction of at least 40%, interruption of long-term beta-blocker treatment was not found to be non-inferior to a strategy of beta-blocker continuation.^32^ Our study, being different from above studies, is an observational study from a real-world acute myocardial infarction population. Furthermore, GDMT refers to medication recommendations according to guidelines, but reflects in this study real-world prescription patterns and is not intended to imply uniform applicability across all patients. A limitation is the lack of left ventricular ejection fraction, as a part of inherent limitation of an administrative database, which does not permit to determine the heart failure phenotype (e.g. between heart failure with reduced ejection fraction and heart failure with preserved ejection fraction). Moreover because of lack of clinical data we were unable to assess the haemodynamic status at the time of beta-blocker initiation. Furthermore, we could not adjudicate the diagnosis of heart failure on an individual level at the time beta-blockers were prescribed. Consequently, our findings should be interpreted with caution and considered only as hypothesis-generating.

Furthermore, persons with a myocardial infarction constitute a heterogenous group, representing patients with varying infarctions sizes and varying frequency of comorbidities. Myocardial infarction was not divided by type in this study, which might also affect our results on beta-blockers considering that there is greater evidence in favour for long-term treatment of beta-blockers in ST-elevated myocardial infarction.^33^ The effect of blocking of beta-adrenergic receptors may vary, with respect to cardiac and metabolic effects, such as negative chronotropic and inotropic effects with lowering of oxygen demand of the heart muscle,^2^ but will also be influenced by the results of interventions that open occlusions and restore oxygen delivery. Beta-blockers have a well-defined treatment recommendation in various cardiovascular syndromes, such as angina pectoris, heart failure with reduced ejection fraction and hypertension,^34,35^ and will be most likely prescribed to a greater extend for patients with these diagnoses. Although, adjustment was performed for comorbidities, possible confounding cannot be fully eliminated and participants with higher risk might receive more medical treatment which might explain our results of slightly higher risk of study endpoints with beta-blocker treatment. Future studies that examine some of these issues will hopefully contribute to developing more specific pharmacological recommendations.^36,37^

Moreover, individuals who adhere to their prescribed medication regimens may constitute a self-selecting group characterized by higher levels of health awareness, education, and motivation to engage in health-promoting behaviours. While statistical adjustments were made for several concurrent medical conditions, it was not feasible to control for all aspects of a healthy lifestyle—such as physical activity, dietary habits, or health literacy—due to limited data availability. As a result, it is possible that the observed associations are partially influenced by a ‘healthy adherer effect’ where better outcomes are driven not solely by the medication but by the overall healthier behaviour of the adherent individuals. This potential bias should be acknowledged when interpreting the findings.

Furthermore, we investigated the cumulative use of each drug group and its association with outcomes. For instance, we found that the magnitude of risk reduction of all-cause mortality and cardiovascular mortality with lipid lowering treatment decreased slightly with longer treatment. This finding is in contrast with previous studies^38^ showing that lowering low-density lipoprotein (LDL) reduces all-cause mortality and major vascular events with more benefits with prolonged treatment. The beneficial effects of lipid lowering treatment depend on the individual risk for an event and the reduction of LDL-cholesterol. Of note, there were no data on individual LDL-levels. Still, most individuals kept using lipid lowering treatment as seen in *Figure 1*, which is expected to have an LDL-lowering effect. Nevertheless, the effectiveness of the lipid lowering treatment also depends on achievement of target levels and overall individual risk profile.

Socioeconomic factors seem to affect pharmacological treatment for secondary prevention after a myocardial infarction, with ethnicity, low income and low education that was associated with discontinuation. Similar findings have previously been published,^12,14^ showing the importance of addressing these issues early and supporting the patient to understand the importance of medication, with continuing measures to reduce the out-of-pocket cost for these medications.

Some comorbidities were associated with discontinuation with medication, which might be related to contraindications, for example kidney failure, but also increased intolerance and vulnerability with aging. However, the presence of other comorbidities may also lead to more extensive pharmacological treatment due to more frequent follow-ups and increased contact with healthcare services. It is noteworthy that the increase or decrease of hazard ratios was at times very small and not considered clinically relevant as defined in our study (*Figure 4*).

### Strength and limitations

In this nationwide register study, a near-complete large cohort of patients were included representing the general population. The extended follow-up of up to 16 years and data collection until year 2022 provides a valuable and up to date information of long-term outcomes and secondary prevention for patients with myocardial infarction. However, this study has also limitations. First, the reason for discontinuing a pharmacological treatment was not known, making it difficult to draw conclusions if the discontinuation was for medical reasons or other person-related factors. Also, adherent participants to prescribed medication might represent a selected group of well-informed and compliant patients that undertake a generally healthier lifestyle. Although, adjustment was done for several comorbidities, this was not possible to do for other preventive measures as for example physical activity. A healthy adherer bias can therefore not be excluded. Secondly, the SPDR provides information of collected medical prescriptions only, and an unknown proportion of patients may not have received a prescription at discharge for some reason. Nevertheless, the purpose of this study was partly to explore discontinuation of secondary preventive pharmacological treatment that is indicated for most patients after a myocardial infarction. Thirdly, the investigators cannot fully be sure of the adherence and intake of medications, although continuous withdrawal of prescriptions indicates the intention and willingness to continue the pharmacological treatment. Finally, the lack of critical, clinical variables, such as myocardial infarction subtype, left ventricular ejection fraction, interventions of revascularization and risk factors as smoking are unavailable. These are central to risk stratification and correct result interpretation and might make it difficult to fully interpret some findings in this study, particularly regarding beta-blockers. Yet, the main aim of this project was to study long-term follow-up and coherence of GDMT as a part of secondary prevention after myocardial infarction.

## Conclusion

Pharmacological treatment as part of secondary prevention after an acute myocardial infarction decreased the first years after discharge and remained underutilized during long-term post-myocardial infarction. Usage of antiplatelets or OAC, ACEi or ARB and lipid lowering medications were associated with decreased risk of all-cause mortality or recurrent myocardial infarction, while treatment with beta-blockers did not have a protective effect in this study. Further efforts need to be undertaken to improve persistence to pharmacological treatment after a myocardial infarction to reduce residual risk for cardiovascular events after a myocardial infarction.

## Supplementary Material

qcag050_Supplementary_Data

## Data Availability

Data are available from the sources stated in the paper on request to the data providers, fulfilling legal and regulatory requirements and with permission from the Swedish Ethical Review Authority.
