# Supplementary material for: Pharmacological treatment after a first myocardial infarction in Sweden—long-term adherence and prognosis
Source: Eur Heart J Qual Care Clin Outcomes. 2026 Mar 26;12(5):782–91. doi: 10.1093/ehjqcco/qcag050 (PMC13457943; doi:10.1093/ehjqcco/qcag050)

	HR (95% CI)	p-value
**All-cause mortality or recurrent MI**		
OAC and antiplatelets	0.70 (0.69 - 0.71)	<.0001
Lipid lowering treatment	0.65 (0.64 - 0.67)	<.0001
Betablockers	1.02 (1.00 - 1.04)	0.013
ACEi/ARB	0.81 (0.80 - 0.82)	<.0001
**All-cause mortality**		
OAC and antiplatelets	0.67 (0.66 - 0.68)	<.0001
Lipid lowering treatment	0.60 (0.59 - 0.62)	<.0001
Betablockers	1.07 (1.05 - 1.09)	<.0001
ACEi/ARB	0.74 (0.73 - 0.75)	<.0001
**Cardiovascular mortality**		
OAC and antiplatelets	0.74 (0.72 - 0.76)	<.0001
Lipid lowering treatment	0.66 (0.64 - 0.68)	<.0001
Betablockers	1.10 (1.07 - 1.13)	<.0001
ACEi/ARB	0.85 (0.83 - 0.87)	<.0001
**Recurrent MI**		
OAC and antiplatelets	0.88 (0.85 - 0.90)	<.0001
Lipid lowering treatment	0.80 (0.77 - 0.82)	<.0001
Betablockers	1.01 (0.99 - 1.04)	0.31
ACEi/ARB	0.97 (0.94 - 0.99)	0.0072

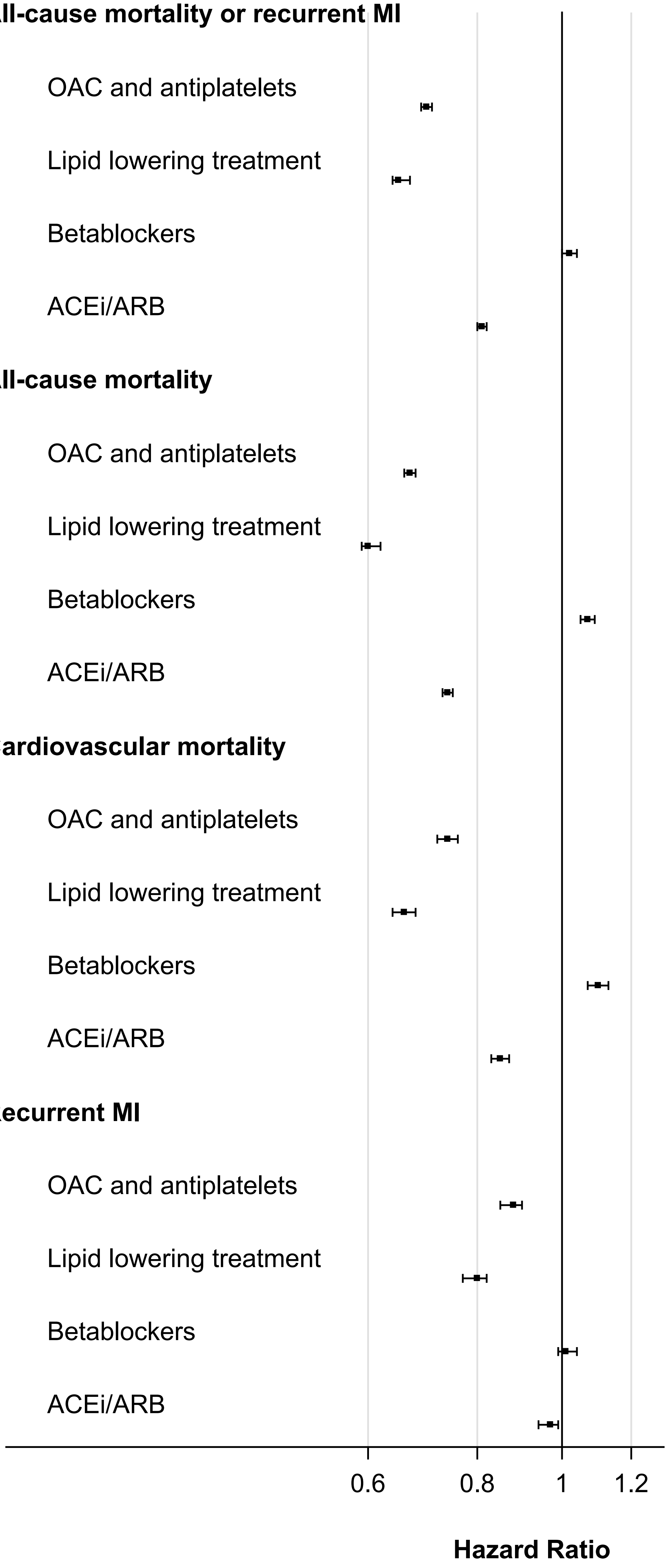

Supplement: Supplementary file 1 [file qcag050_supplementary_data.zip › Supp.Figure_01-250711.pdf]
