# Supplementary material for: Pharmacological treatment after a first myocardial infarction in Sweden—long-term adherence and prognosis
Source: Eur Heart J Qual Care Clin Outcomes. 2026 Mar 26;12(5):782–91. doi: 10.1093/ehjqcco/qcag050 (PMC13457943; doi:10.1093/ehjqcco/qcag050)
Supplement: Supplementary file 1 [file qcag050_supplementary_data.zip › SUPPLEMENTARY-260330.docx]

**SUPPLEMENTARY**

Supplementary Table 1. Drug classes and their Anatomic Therapeutic Chemical classification.

| Drug class | ATC code |
| --- | --- |
| **Lipid lowering therapy** |  |
| Statin | C10AA  C10BA02  C10BA05 |
| Ezetimibe | C10AX09 |
| PCSK9-inhibitors | C10AX13  C10AX14 |
| **Antiplatelet and oral anticoagulant therapy** |  |
| Antiplatelet | B01AC |
| Warfarin | B01AA |
| Eliquis, Xarelto, Lixiana | B01AF |
| Pradaxa | B01AE07 |
| **ACEi/ARB** | C09 |
| **Betablocker** | C07, excl C07AA07 |

ATC = anatomic chemical classification, PCSK9 = protein convertase subtilisin/kexin type 9, ACEi = angiotensin converting enzyme inhibitor, ARB = angiotensin receptor blocker

Supplementary Table 2. The international classification of disease, ICD-9 and ICD-10.

The following ICD-codes were acquired.

| **Disease** | **ICD-9** | **ICD-10** |
| --- | --- | --- |
| Hypertension | 401-405 | I10-I15 |
| Diabetes mellitus | 250 | E10-14 |
| Heart failure | 428 | I50 |
| Atrial fibrillation | 427.D, 427.3 | I48 |
| Stroke | 431-434, 436 | I61-I64, I69 |
| Chornic respiratory disease/Asthma | 490-496 | J40-J47 |
| Peripheral vascular disease | 440, 441, 442, 443, 444, 447 | I65-I65.9, I70-I74, I77 |
| Kidney failure | 584-586 | N17-N19 |
| Adult congenital heart disease | 745-747 | Q20-Q26 |
| Cancer | 140-208 | C00-C97 |
| Valvular disease | 394-397, 424 | I05-I08, I34-I37 |

Supplementary Table 3. Patient demographics per medication discontinuation during first year

| **Variable** | **Discontinuation of Lipid Lowering treatment within 1 year N=16493** | **Discontinuation of OAC/Antiplatelets within 1 year N=11384** | **Discontinuation of ACEi/ARB within 1 year N=14329** | **Discontinuation of Betablockers within 1 year N=17435** |
| --- | --- | --- | --- | --- |
| ***Patient demographics and socioeconomic status*** |  |  |  |  |
| Age (years) | 66.7±10.7 68 (18 - 84) n=16493 | 66.1±11.1 67 (20 - 84) n=11384 | 67.2±10.5 68 (18 - 84) n=14329 | 66.2±10.8 67 (18 - 84) n=17435 |
| Age category (years) |  |  |  |  |
| <65 years | 6524 (39.6%) | 4792 (42.1%) | 5335 (37.2%) | 7256 (41.6%) |
| 65-74 years | 5634 (34.2%) | 3671 (32.2%) | 4985 (34.8%) | 5779 (33.1%) |
| 75-84 years | 4335 (26.3%) | 2921 (25.7%) | 4009 (28.0%) | 4400 (25.2%) |
| Country of birth |  |  |  |  |
| Nordic countries | 14258 (86.5%) | 9708 (85.3%) | 12298 (85.8%) | 15113 (86.7%) |
| Non-Nordic countries | 2232 (13.5%) | 1670 (14.7%) | 2027 (14.2%) | 2316 (13.3%) |
| Missing | 3 | 6 | 4 | 6 |
| Marital status |  |  |  |  |
| Married/Cohabiting | 9244 (56.0%) | 6315 (55.5%) | 8094 (56.5%) | 9994 (57.3%) |
| Not married | 2502 (15.2%) | 1733 (15.2%) | 2154 (15.0%) | 2735 (15.7%) |
| Divorced | 3293 (20.0%) | 2318 (20.4%) | 2820 (19.7%) | 3324 (19.1%) |
| Widowed | 1435 (8.7%) | 993 (8.7%) | 1242 (8.7%) | 1358 (7.8%) |
| Unknown | 19 (0.1%) | 25 (0.2%) | 19 (0.1%) | 24 (0.1%) |
| Education (years) |  |  |  |  |
| <10 | 5188 (32.0%) | 3659 (32.9%) | 4428 (31.5%) | 5267 (30.7%) |
| 10-12 | 7201 (44.4%) | 4912 (44.1%) | 6344 (45.1%) | 7681 (44.8%) |
| >12 | 3821 (23.6%) | 2561 (23.0%) | 3302 (23.5%) | 4200 (24.5%) |
| Missing | 283 | 252 | 255 | 287 |
| Income |  |  |  |  |
| Q1 (lowest) | 2094 (12.7%) | 1591 (14.0%) | 1832 (12.8%) | 1998 (11.5%) |
| Q2 | 2404 (14.6%) | 1672 (14.7%) | 2053 (14.3%) | 2312 (13.3%) |
| Q3 | 3152 (19.1%) | 2134 (18.8%) | 2704 (18.9%) | 3170 (18.2%) |
| Q4 | 3991 (24.2%) | 2697 (23.7%) | 3562 (24.9%) | 4291 (24.6%) |
| Q5 (highest) | 4831 (29.3%) | 3265 (28.7%) | 4158 (29.1%) | 5640 (32.4%) |
| Missing | 21 | 25 | 20 | 24 |
| ***Medical history before incident MI diagnosis*** |  |  |  |  |
| Diabetes | 3014 (18.3%) | 1984 (17.4%) | 2851 (19.9%) | 2847 (16.3%) |
| Hypertension | 7902 (47.9%) | 5281 (46.4%) | 7534 (52.6%) | 8016 (46.0%) |
| Heart failure | 1775 (10.8%) | 1363 (12.0%) | 1718 (12.0%) | 1794 (10.3%) |
| Atrial fibrillation | 1679 (10.2%) | 1238 (10.9%) | 1573 (11.0%) | 1729 (9.9%) |
| Stroke | 949 (5.8%) | 650 (5.7%) | 894 (6.2%) | 897 (5.1%) |
| Chronic respiratory disease/asthma | 1611 (9.8%) | 1220 (10.7%) | 1464 (10.2%) | 1624 (9.3%) |
| Peripheral vascular disease | 1143 (6.9%) | 764 (6.7%) | 1067 (7.4%) | 1069 (6.1%) |
| Kidney failure | 675 (4.1%) | 496 (4.4%) | 771 (5.4%) | 676 (3.9%) |
| Congenital heart disease | 46 (0.3%) | 41 (0.4%) | 41 (0.3%) | 43 (0.2%) |
| Cancer | 2419 (14.7%) | 1671 (14.7%) | 2200 (15.4%) | 2546 (14.6%) |
| Valvular disease | 682 (4.1%) | 536 (4.7%) | 695 (4.9%) | 718 (4.1%) |
| Myocardial infarction | 0 (0%) | 0 (0%) | 0 (0%) | 0 (0%) |
| ***Medications before incident MI diagnosis*** |  |  |  |  |
| OAC and antiplatelets | 4224 (25.6%) | 2968 (26.1%) | 3982 (27.8%) | 4315 (24.7%) |
| Lipid lowering treatment | 3647 (22.1%) | 2460 (21.6%) | 3422 (23.9%) | 3766 (21.6%) |
| ß-blockers | 4296 (26.0%) | 2943 (25.9%) | 3932 (27.4%) | 4534 (26.0%) |
| ACEi/ARB | 5270 (32.0%) | 3519 (30.9%) | 5687 (39.7%) | 5356 (30.7%) |
| Data are presented as mean±standard deviation, median (range) and number of observations, or number (%).One and the same patient might be included in more than one medication group. OAC = oral anticoagulant, ACEi = angiotensin converting enzyme inhibitor, ARB = angiotensin receptor blocker | | | | |

Supplementary Table 4. Follow-up time and event rates of studied endpoints.

| **Endpoint** | **n (%) events** | **Follow-up time Median (IQR)** | **Event rate (95% CI)** |
| --- | --- | --- | --- |
| **All-cause mortality** | 31652 (32.9%) | 5.61 (1.85-10.55) Sum=620135 | 5.10 (5.05-5.16) |
| **Recurrent MI** | 17128 (17.8%) | 4.01 (0.51-9.24) Sum=513752 | 3.33 (3.28-3.38) |
| **All-cause mortality or recurrent MI** | 42202 (43.8%) | 4.01 (0.51-9.24) Sum=513752 | 8.21 (8.14-8.29) |
| **Cardiovascular mortality** | 16873 (17.5%) | 5.61 (1.85-10.55) Sum=620070 | 2.72 (2.68-2.76) |
| Confidence interval for event rates per 100 person years are obtained from exact Poisson confidence limits. MI = myocardial infarction | | | |

|  | **Time-updated use of lipid lowering treatment, antiplatelets/OAC, ACEi/ARB** | | **Time-updated use of lipid lowering treatment, antiplatelets/OAC, ACEi/ARB, betablockers** | |
| --- | --- | --- | --- | --- |
| **Model** | **HR (95% CI)** | **p-value** | **HR (95% CI)** | **p-value** |
| **All-cause mortality or recurrent MI** | 0.57 (0.55 - 0.58) | <.0001 | 0.58 (0.57 - 0.60) | <.0001 |
| **All-cause mortality** | 0.52 (0.50 - 0.53) | <.0001 | 0.54 (0.52 - 0.56) | <.0001 |
| **Cardiovascular mortality** | 0.62 (0.60 - 0.64) | <.0001 | 0.66 (0.63 - 0.69) | <.0001 |
| **Recurrent MI** | 0.76 (0.73 - 0.80) | <.0001 | 0.78 (0.74 - 0.81) | <.0001 |
| Adjusted for: OAC and antiplatelets (unless main variable), Lipid lowering treatment (unless main variable), betablockers (unless mail variable), ACE inhibitor/ARB (unless main variable), calendar year, Nordic/non-Nordic countries, marital status, education, income, diabetes, hypertension, heart failure, atrial fibrillation, stroke, chronic respiratory disease/asthma, peripheral vascular  disease, kidney failure, congenital heart disease, cancer, valvular disease. OAC = oral anticoagulant, ACEi = angiotensin converting enzyme inhibitor, ARB = angiotensin receptor blocker inhibitor, MI = myocardial infarction | | | | |

Supplementary Table 5. Adjusted effects of time-updated medications for all-cause mortality, recurrent myocardial infarction and cardiovascular mortality in individuals with myocardial infarction 2006-2022.

Supplementary Table 6.1. Cumulative significance of compliance to medication by grouping years of use for all-cause mortality using Cox regression.

|  | **Time-updated use of OAC and antiplatelets** | | **Time-updated use of lipid lowering treatment** | | **Time-updated use of betablockers** | | **Time-updated use of ACEi/ARB** | |
| --- | --- | --- | --- | --- | --- | --- | --- | --- |
| **Duration of medication use compared to no use** | **HR (95% CI)** | **p-value** | **HR (95% CI)** | **p-value** | **HR (95% CI)** | **p-value** | **HR (95% CI)** | **p-value** |
| **<1 year** | 0.39 (0.36 - 0.42) | <.0001 | 0.45 (0.42 - 0.49) | <.0001 | 1.08 (1.03 - 1.13) | 0.46 | 0.82 (0.79 - 0.86) | 0.025 |
| **1-<5 years** | 0.09 (0.08 - 0.10) | <.0001 | 0.43 (0.38 - 0.48) | 0.0002 | 1.75 (1.65 - 1.85) | <.0001 | 0.71 (0.68 - 0.74) | 0.0003 |
| **5-<10 years** | 0.02 (0.02 - 0.02) | <.0001 | 0.48 (0.42 - 0.54) | 0.0052 | 2.01 (1.86 - 2.18) | <.0001 | 0.51 (0.48 - 0.54) | <.0001 |
| **≥ 10 years** | 0.00 (0.00 - 0.00) | <.0001 | 0.49 (0.41 - 0.59) | 0.054 | 2.07 (1.75 - 2.46) | 0.035 | 0.61 (0.52 - 0.72) | 0.13 |
| Adjusted for: OAC and antiplatelets (unless main variable), Lipid lowering treatment (unless main variable), beta-blockers (unless mail variable), ACEi/ARB (unless main variable), calendar year, nordic/non-nordic countries, marital status, education, income, diabetes, hypertension, heart failure, atrial fibrillation, stroke, chronic respiratory disease/asthma, peripheral vascular disease, kidney failure, congenital heart disease, cancer, valvular disease.  OAC = oral anticoagulant, ACEi = angiotensin converting enzyme inhibitor, ARB = angiotensin receptor blocker | | | | | | | | |

Supplementary Table 6.2. Cumulative significance of compliance to medication by grouping years of use for myocardial infarction using Cox regression.

|  | **Time-updated use of OAC and antiplatelets** | | **Time-updated use of lipid lowering treatment** | | **Time-updated use of betablockers** | | **Time-updated use of ACEi/ARB** | |
| --- | --- | --- | --- | --- | --- | --- | --- | --- |
| **Duration of medication use compared to no use** | **HR (95% CI)** | **p-value** | **HR (95% CI)** | **p-value** | **HR (95% CI)** | **p-value** | **HR (95% CI)** | **p-value** |
| **<1 year** | 0.45 (0.41 - 0.48) | <.0001 | 0.93 (0.86 - 1.00) | 0.62 | 1.16 (1.09 - 1.24) | 0.25 | 1.30 (1.22 - 1.38) | 0.031 |
| **1-<5 years** | 0.25 (0.21 - 0.30) | <.0001 | 1.45 (1.22 - 1.74) | 0.30 | 1.22 (1.11 - 1.35) | 0.31 | 0.62 (0.57 - 0.67) | 0.0045 |
| **5-<10 years** | 0.14 (0.11 - 0.18) | 0.0001 | 0.32 (0.25 - 0.42) | 0.029 | 0.87 (0.74 - 1.03) | 0.68 | 0.53 (0.46 - 0.61) | 0.029 |
| **≥ 10 years** | 0.14 (0.09 - 0.23) | 0.046 | 0.10 (0.07 - 0.16) | 0.0069 | 0.69 (0.47 - 1.03) | 0.65 | 0.52 (0.36 - 0.76) | 0.40 |
| Adjusted for: OAC and antiplatelets (unless main variable), Lipid lowering treatment (unless main variable), beta-blockers (unless mail variable), ACEi/ARB (unless main variable), calendar year, nordic/non-nordic countries, marital status, education, income, diabetes, hypertension, heart failure, atrial fibrillation, stroke, chronic respiratory disease/asthma, peripheral vascular disease, kidney failure, congenital heart disease, cancer, valvular disease.  OAC = oral anticoagulant, ACEi = angiotensin converting enzyme inhibitor, ARB = angiotensin receptor blocker | | | | | | | | |

|  | **Time-updated use of OAC and antiplatelets** | | **Time-updated use of lipid lowering treatment** | | **Time-updated use of betablockers** | | **Time-updated use of ACEi/ARB** | |
| --- | --- | --- | --- | --- | --- | --- | --- | --- |
| **Duration of medication use compared to no use** | **HR (95% CI)** | **p-value** | **HR (95% CI)** | **p-value** | **HR (95% CI)** | **p-value** | **HR (95% CI)** | **p-value** |
| **<1 year** | 0.29 (0.28 - 0.31) | <.0001 | 0.78 (0.73 - 0.82) | 0.025 | 0.96 (0.92 - 1.00) | 0.60 | 0.95 (0.92 - 0.99) | 0.53 |
| **1-<5 years** | 0.08 (0.08 - 0.09) | <.0001 | 0.70 (0.64 - 0.78) | 0.08 | 1.35 (1.28 - 1.43) | 0.0067 | 0.68 (0.65 - 0.71) | <.0001 |
| **5-<10 years** | 0.02 (0.02 - 0.02) | <.0001 | 0.65 (0.58 - 0.74) | 0.09 | 1.43 (1.32 - 1.55) | 0.025 | 0.52 (0.49 - 0.56) | <.0001 |
| **≥ 10 years** | 0.00 (0.00 - 0.00) | <.0001 | 0.52 (0.43 - 0.63) | 0.08 | 1.52 (1.27 - 1.82) | 0.26 | 0.76 (0.64 - 0.90) | 0.42 |
| Adjusted for: OAC and antiplatelets (unless main variable), Lipid lowering treatment (unless main variable), beta-blockers (unless mail variable), ACEi/ARB (unless main variable), calendar year, nordic/non-nordic countries, marital status, education, income, diabetes, hypertension, heart failure, atrial fibrillation, stroke, chronic respiratory disease/asthma, peripheral vascular disease, kidney failure, congenital heart disease, cancer, valvular disease.  OAC = oral anticoagulant, ACEi = angiotensin converting enzyme inhibitor, ARB = angiotensin receptor blocker | | | | | | | | |

Supplementary Table 6.3. Cumulative significance of compliance to medication by grouping years of use for all-cause mortality or recurrent myocardial infarction using Cox regression.

Supplementary Table 6.4. Cumulative significance of compliance to medication by grouping years of use for cardiovascular mortality using Cox regression.

|  | **Time-updated use of OAC and antiplatelets** | | **Time-updated use of lipid lowering treatment** | | **Time-updated use of betablockers** | | **Time-updated use of ACEi/ARB** | |
| --- | --- | --- | --- | --- | --- | --- | --- | --- |
| **Duration of medication use compared to no use** | **HR (95% CI)** | **p-value** | **HR (95% CI)** | **p-value** | **HR (95% CI)** | **p-value** | **HR (95% CI)** | **p-value** |
| **<1 year** | 0.43 (0.39 - 0.47) | <.0001 | 0.34 (0.31 - 0.37) | <.0001 | 1.25 (1.17 - 1.34) | 0.11 | 1.19 (1.12 - 1.26) | 0.17 |
| **1-<5 years** | 0.10 (0.08 - 0.11) | <.0001 | 0.24 (0.21 - 0.28) | <.0001 | 2.44 (2.23 - 2.68) | <.0001 | 1.35 (1.25 - 1.45) | 0.049 |
| **5-<10 years** | 0.02 (0.01 - 0.02) | <.0001 | 0.28 (0.23 - 0.34) | 0.0013 | 2.65 (2.34 - 3.00) | 0.0001 | 0.94 (0.84 - 1.04) | 0.76 |
| **≥ 10 years** | 0.00 (0.00 - 0.00) | <.0001 | 0.37 (0.28 - 0.49) | 0.08 | 2.62 (1.99 - 3.45) | 0.08 | 0.80 (0.62 - 1.03) | 0.67 |
| Adjusted for: OAC and antiplatelets (unless main variable), Lipid lowering treatment (unless main variable), beta-blockers (unless mail variable), ACEi/ARB (unless main variable), calendar year, nordic/non-nordic countries, marital status, education, income, diabetes, hypertension, heart failure, atrial fibrillation, stroke, chronic respiratory disease/asthma, peripheral vascular disease, kidney failure, congenital heart disease, cancer, valvular disease.  OAC = oral anticoagulant, ACEi = angiotensin converting enzyme inhibitor, ARB = angiotensin receptor blocker | | | | | | | | |
